# An unusual cause of epigastric pain: Gastric syphilis

**DOI:** 10.1002/jgf2.712

**Published:** 2024-06-24

**Authors:** Naoya Itoh, Shun Iida, Masahiro Tajika

**Affiliations:** ^1^ Division of Infectious Diseases Aichi Cancer Center Hospital Nagoya Aichi Japan; ^2^ Department of Infectious Diseases, Graduate School of Medical Sciences Nagoya City University Nagoya Aichi Japan; ^3^ Department of Infectious Diseases Nagoya City University East Medical Center Nagoya Aichi Japan; ^4^ Department of Pathology National Institute of Infectious Diseases Tokyo Japan; ^5^ Department of Endoscopy Aichi Cancer Center Hospital Nagoya Aichi Japan

**Keywords:** infectious diseases

## Abstract

(A) Gastroscopy showing multiple geographic erosions with white plaques from the angulus to the antrum. (B) Immunohistochemical staining for *Treponema pallidum* following gastric biopsy showing an abundance of brown syphilis spirochetes.

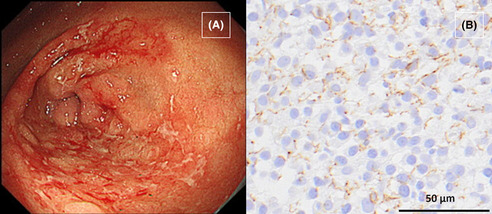

A 46‐year‐old Japanese woman presented to our clinic with epigastric pain lasting for 2 months. Two months prior, she had experienced transient erythema on her abdomen and axillae, which resolved spontaneously. During a detailed interview, she reported a history of genital herpes a month prior and stated that her partner had been treated for syphilis 2 weeks prior. She had been in a relationship with the partner for 10 months and had no other sexual contact with other partners. She is unemployed and lives with her father, and she denied engaging in commercial sex work. Physical examination revealed epigastric tenderness. Serum *Treponema pallidum* antibody testing was positive, and the rapid plasma reagin (RPR) titer was 1:128. However, her human immunodeficiency virus test was negative. Gastroscopy revealed erosions with white plaques from the angulus to the antrum (Figure 1A). Gastric biopsies were performed, and subsequent histopathological analysis revealed the formation of granulation tissue and severe inflammatory infiltration by lymphocytes and plasma cells, without any features of malignancy. Immunohistochemistry (Figure 1B) and real‐time polymerase chain reaction (PCR) all indicated the presence of *T. pallidum* in the gastric tissues. The final diagnosis was gastric syphilis, and the patient was treated with a single dose of intramuscular benzathine penicillin G (2.4 million units), which resolved her clinical complaints. Gastroscopy performed 3 months after treatment showed prominent improvement of inflammatory changes observed in the angulus to the antrum (Figure 2). At that time, her RPR titer had decreased to 1:8.

**FIGURE 1:**
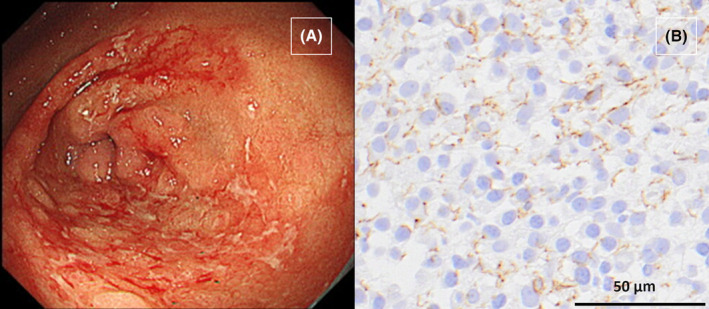
(A) Gastroscopy showing multiple geographic erosions with white plaques from the angulus to the antrum. (B) Immunohistochemical staining for *Treponema pallidum* following gastric biopsy showing an abundance of brown syphilis spirochetes.

**FIGURE 2:**
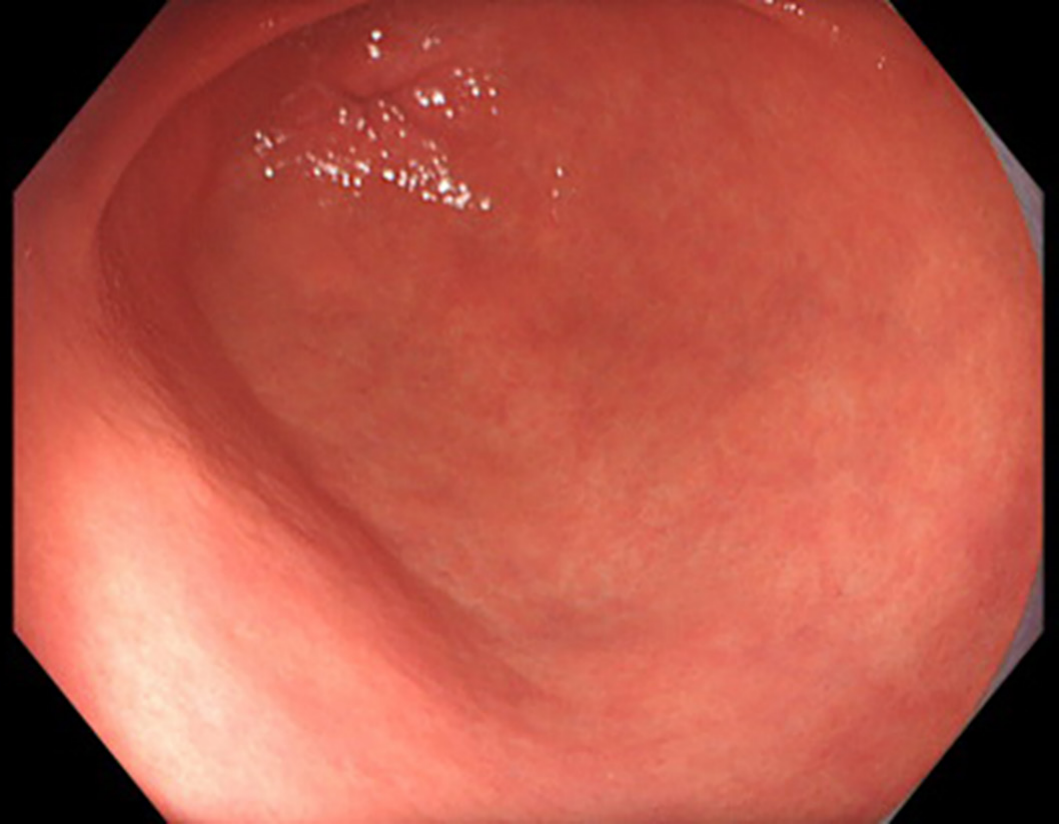
Gastroscopy after treatment, demonstrating significant improvement of inflammatory changes from the angulus to the antrum.

Although the incidence of syphilis is increasing worldwide, gastric syphilis is observed in only 1% of infected patients.^1^ Although it can occur at any stage, approximately 50% of these cases are associated with secondary syphilis.^2^ Our case was treated with a single dose of benzathine penicillin G, a second‐stage syphilis treatment, as the infection likely occurred within 1 year from her current partner.^3^ The presentation of gastric syphilis includes nonspecific gastrointestinal symptoms,^2^ while gastroscopy typically reveals diminished gastric expandability, accompanied by erosions, ulcers, mucosal edema, thickened gastric folds, nodularity, masses, and hypertrophy of the gastric folds.^4^ Histologically, gastric syphilis is characterized by a prominent infiltration of lymphohistiocytic cells, significant numbers of plasma cells, and lymphoid aggregates, all accompanied by acute inflammation.^3^ Thus, gastric syphilis can clinically and histologically resemble other gastric conditions, including lymphoproliferative diseases, invasive carcinomas, tuberculosis, and Crohn's disease. Definitive diagnosis is made by PCR testing for *T. pallidum* in frozen and paraffin‐embedded tissues, with immunohistochemical staining for *T. pallidum*‐specific antigens. Given its treatable nature, clinicians must recognize gastric syphilis swiftly to avoid unnecessary tests and treatments.

## AUTHOR CONTRIBUTIONS

Naoya Itoh was involved in the literature review, study planning, and manuscript writing. Naoya Itoh and Masahiro Tajika were involved in patient care. Shun Iida conducted laboratory analyses. All authors interpreted the data and drafted and critically revised the manuscript, approving its final version. All authors meet the ICMJE authorship criteria.

## FUNDING INFORMATION

This study did not receive any specific grants from funding agencies in the public, commercial, or not‐for‐profit sectors.

## CONFLICT OF INTEREST STATEMENT

The authors have stated explicitly that there are no conflicts of interest in connection with this article.

## ETHICS STATEMENT

Ethics approval statement: None.

Patient consent statement: None.

Clinical trial registration: None.

## CONSENT FOR PUBLICATION

The patient provided written informed consent for the publication of this report and the accompanying images.

## Data Availability

All relevant data are within the paper.
